# Unexpected Case of Cardiac Sarcoidosis in a Caucasian Male

**DOI:** 10.7759/cureus.33353

**Published:** 2023-01-04

**Authors:** Stacey Damito, Zhongying Liu-An

**Affiliations:** 1 Internal Medicine, Hackensack University Medical Center, Hackensack, USA

**Keywords:** corticosteroids, caucasian, demographic characteristics, sarcoidosis, cardiac sarcoidosis

## Abstract

Cardiac sarcoidosis (CS) is an underappreciated diagnosis in healthy patients presenting with recurrent syncope. This may be particularly limited in patients who do not meet common epidemiology and manifestations of sarcoidosis, which are typically African American women and pulmonary, respectively. In our case, we have a previously healthy middle-aged Caucasian American male who presented with recurrent syncope for one week. Initial electrocardiogram showed a right bundle branch block with a normal P-R interval. A few days into the admission, the patient suffered another episode of syncope precipitated by micturition, and repeat electrocardiogram revealed evolution to complete atrioventricular block, necessitating emergent placement of a temporary permanent pacemaker. Transthoracic echocardiogram showed preserved left ventricular ejection fraction of 55%-60% with normal heart valves. Chest computerized tomography revealed few pulmonary nodules, prompting a weak concern for infiltrative disease, e.g., sarcoidosis. To evaluate for possible cardiac structural abnormalities, a cardiac magnetic resonance imaging (MRI) study was considered but precluded by the presence of MRI-incompatible temporary pacemaker. Despite low suspicion, a fluorodeoxyglucose-positron emission tomography was obtained which unexpectedly revealed hypermetabolic lymph nodes in the perihilar, supraclavicular, and mediastinal regions as well as an area along the interventricular septum, consistent with atrioventricular (AV) conduction pathways. As the patient met major criteria for CS per Japanese Circulation Society guidelines, a tentative diagnosis was made, and a Biotronik single-chamber implantable cardiac defibrillator was ultimately placed. On outpatient follow-up, endobronchial ultrasound-guided fine-needle biopsy of perihilar lymph nodes revealed only rare epithelioid histiocytes, rare alveolar macrophages, and benign bronchial cells, consistent with benign nodal tissue. Further attempts for histological confirmation were aborted due to profound calcification and location of affected lymph nodes. A decision was made to defer further biopsy, including the gold standard of diagnosis endomyocardial biopsy, due to the risks outweighing the benefits. He initiated medical therapy with prednisone and mycophenolate, as well as trimethoprim-sulfamethoxazole for Pneumocystis prophylaxis. Unlike general sarcoidosis, which is often considered a benign systemic disease, CS has high potential for severe complications including arrhythmia, systolic heart failure, and sudden cardiac death. In general, males carry a higher risk of CS than females, especially those who are of African American descent as they carry a higher incidence of nonspecific sarcoidosis. Expectations related to our patient’s demographic initially delayed diagnostic workup for infiltrative disease, primarily focusing on intracranial, orthostatic, and infectious causes. This case report serves to inform clinicians on early manifestations of CS, raise awareness of its incidence in unexpected demographics, and encourage them to consider infiltrative diseases when presented with patients of similar symptoms.

## Introduction

Cardiac sarcoidosis (CS) has become an increasingly recognized diagnosis in patients with new and unexpected cardiac dysfunctions [1]. While sarcoidosis is largely known as a benign systemic disease, it remains enigmatic given its heterogeneous presentation and lack of reliable screening tests [2]. It is most notable for its pulmonary and skin manifestations, but almost 30% of patients are shown to also have cardiac involvement [3]. The pathophysiology of CS includes granuloma formation and inflammatory infiltrate of cardiac tissue resulting in conduction abnormalities, ventricular dysfunction, and cardiomyopathy. Current studies based on imaging and autopsies over the last couple of decades have revealed that about 25% of these cases in particular are isolated CS (ICS), an underappreciated phenotype characterized by an apparent absence of extracardiac findings [2,4]. This unique presentation may often preclude a differential diagnosis of infiltrative disease when assessing a patient with new onset arrhythmia or systolic heart failure. However, with increasing awareness of CS, as well as growing input on appropriate diagnostic modalities and guidelines, we are now able to achieve early diagnosis and subsequently lifesaving interventions [1].

The diagnosis of CS is guided by three major societies: the Heart Rhythm Society 2014 [5], Japanese Circulation Society 2017 [6], and World Association of Sarcoidosis and Other Granulomatous Diseases (WASOG) 2014 [7]. While histological confirmation offers the highest level of certainty, the gold standard for diagnosis, endomyocardial biopsy (EMB), still carries limited sensitivity that is estimated to be around 25% [8]. Therefore, all three guidelines provide room for clinical diagnosis supported by a preferred imaging modality, e.g., 18F‐fluorodeoxyglucose positron emission tomography (FDG-PET) or late gadolinium enhancement on cardiac magnetic resonance imaging (LGE on CMR); electrocardiogram; and echocardiogram [1,3].

On an epidemiological note, sarcoidosis is classically associated with a higher prevalence in Nordic countries and African Americans (AAs), and it appears to affect women more than men [2,9]. Data also show that while Caucasian American men, who are already affected three times less than AA men, are often asymptomatic upon diagnosis, their AA counterparts frequently suffer higher mortality related to the disease [2]. In clinical practice, these epidemiological patterns may be familiar to a general internist. While in most cases, this may favor quick management of patients who present with the expected symptoms and demographics, it can carry the risk of overlooking potential diagnoses in atypical cases. In our case, we encountered an otherwise healthy White American man who presented with recurrent syncope and was found to have an unexpected diagnosis of CS.

## Case presentation

A previously healthy 51-year-old Caucasian American male presents to the hospital with multiple episodes of syncope for one week. He also notes that he has had intermittent orthostatic dizziness for the past few months. These syncopal episodes appear to be triggered by positional changes like bending forward and vasovagal maneuvers like urinating. In his initial presentation to the emergency department, electrocardiogram reveals a right bundle branch block with a normal P-R interval. Transthoracic echocardiogram (TTE) shows a left ventricular ejection fraction (LVEF) of 55%-60% with normal heart valves. Despite a benign workup, he presents to the hospital multiple times over the week and is eventually admitted for closer monitoring. One night into hospitalization, the patient is sitting on the toilet when he loses consciousness. Telemetry reveals a new complete atrioventricular block with pauses lasting up to 11 seconds. An electrophysiologist is emergently consulted, and a temporary permanent pacemaker is placed in the cardiac catheterization lab per 2018 American Heart Association guidelines [10].

**Figure 1:**
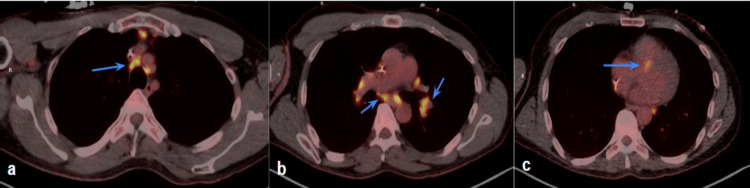
PET-CT chest shows hypermetabolic activity among cardiac and mediastinal structures. (a) mediastinal lymph nodes, (b) perihilar lymph nodes, and (c) interventricular septum. PET-CT: positron emission tomography-computed tomography

**Figure 2:**
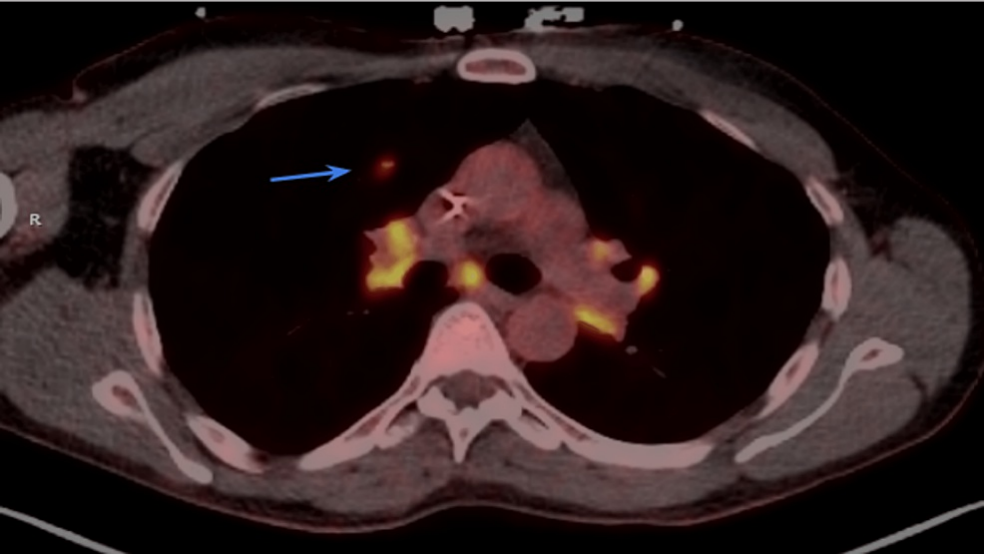
PET-CT chest of hypermetabolic activity in a pulmonary nodule, suggestive of infiltrative disease like sarcoidosis. PET-CT: positron emission tomography-computed tomography

Workup for other causes of syncope also reveals a few pulmonary nodules, measuring up to 14 mm, on computerized tomography (CT), raising suspicion for sarcoidosis. The pulmonology team is consulted, who notes that his nodular distribution is not consistent with typical pulmonary sarcoidosis given scarcity and location. On further history-taking, the patient denies any personal or family history of dyspnea, cough, or other classic manifestations of sarcoidosis or other inflammatory diseases. While a diagnostic cardiac magnetic resonance imaging (MRI) study is desired, the patient is unable to complete one due to the presence of his non-MRI-compatible temporary pacemaker. The patient instead undergoes a FDG-PET, which shows areas of hypermetabolism in lymph nodes of the supraclavicular region, mediastinum, and hila (Figures 1a-1b), as well as small right lung pulmonary nodules (Figure 2). More interestingly, it showed areas of high metabolic activity along the interventricular septum, posing a viable etiology for the patient’s atrioventricular dysfunction (Figure 1c). Given high suspicion of infiltrative disease, a decision was made to place a Biotronik single-chamber implantable cardiac defibrillator (ICD), which the patient tolerated well. He did not have any further cardiac or syncopal events during his brief hospitalization, and he was discharged home by the next day for outpatient electrophysiology and pulmonology follow-up.

To diagnose his disease by pathology, a supraclavicular nodule biopsy was initially attempted by interventional radiology while the patient was still inpatient. Upon visualization, it was noted that the location of the desired specimen was in a high risk location given its proximity to other vital structures. The procedure was aborted, and workup was deferred to outpatient follow-up. With the pulmonology team, an outpatient endobronchial ultrasound (EBUS)-guided fine needle biopsy of the right hilar and subcarinal lymph nodes was performed. Pathology was consistent with only benign nodal tissue, e.g. rare epithelioid histiocytes, lymphocytes, benign appearing bronchial cells, alveolar macrophages, and fragments of cartilage, neutrophils and red blood cells. Further attempts for sampling were aborted due to profound calcification of remaining nodules, and discussions regarding pursuit of gold standard diagnostics, i.e. endomyocardial biopsy, were deferred due to risks outweighing benefits.

While biopsy did not help confirm diagnosis, the etiology of lymphadenopathy was unclear. Characteristic presentation, history, and evidence for malignancy and infection were absent. A respiratory pathogen panel was negative for common virology, and flow cytometry showed no evidence of lymphoma. However, while the scarce distribution of affected lymph nodes initially lowered suspicion for pulmonary sarcoidosis, its calcified nature maintained a mild one including that for other infiltrative diseases like amyloidosis. Ultimately, the patient still met two major criteria based on Japanese Circulation Society guidelines, including both presence of high degree AV block and positive uptake on positron emission tomography (PET) imaging consistent with cardiac sarcoidosis [7]. A decision was made to start him on prednisone 40 mg oral daily, mycophenolate 500 mg oral twice daily, and trimethoprim-sulfamethoxazole three times per week for Pneumocystis prophylaxis, with a plan to repeat cardiac PET in three months.

At his two-week follow-up, the patient's ICD was interrogated by electrophysiology, who noted no further episodes of atrial arrhythmias or non-sustained ventricular tachycardia since placement.

## Discussion

The above patient’s case presents multiple areas of interest: unexpected presentation and demographic, and absence of meaningful extracardiac involvement that is verifiable by biopsy. Sarcoidosis can affect any organ, but not all organs are affected equally. According to the American Clinical Characteristics of Patients in a case-control study of sarcoidosis, 95% of cases have pulmonary involvement, while only 2.3% of patients have cardiac involvement [9]. Unlike extracardiac manifestations, which have the benefit of presenting as typically benign symptoms, CS carries a high risk of severe complications including arrhythmia, systolic heart failure, and at worst sudden cardiac death [11]. Multiple studies show that cardiac symptoms can be the first and only manifestations of CS, with AV block being the most common and initial arrhythmic manifestation, especially in middle-aged patients [12-14]. In our case, the patient’s PET scan showed two hypermetabolic areas in the interventricular septum, showing a tracer uptake pattern consistent with granulomatous infiltration; the location of these lesions likely interrupted AV conduction to the point of complete heart block. It is worth acknowledging that without histological evidence, one might consider other infiltrative diseases, like cardiac amyloidosis. Diagnosis was deemed less likely given that the biopsy did not show characteristic fibrillar changes and that the patient did not demonstrate progressive diastolic dysfunction, a hallmark of disease [15]. Malignancy and infection had also been ruled out as he did not present with a contributory history, nor a remarkable diagnostic workup, to support these diagnoses. Despite the results, the patient had the fortunate opportunity to present to the hospital with non-fatal symptoms. Given that the potential severity of a likely diagnosis of CS was clear, the immediate placement of an intracardiac defibrillator allowed him to preempt progression from what was initially pre-syncope in the months leading up to his presentation to potentially sudden cardiac death.

Of additional interest is the disease’s epidemiology, particularly regarding its expected affected demographic. There are reports of racial and gender differences of sarcoidosis in the United States. AA females have the highest incidence of general sarcoidosis, followed by AA males, Caucasian females, and Caucasian males [9]. One learning point that deserves special attention is that males carry a higher risk for cardiac sarcoidosis compared to females [16-17]. Our patient presented as the least commonly affected demographic of sarcoidosis being a Caucasian male. Given that he had an atypical presentation with only cardiac symptoms, his initial workup focused on ruling out intracranial pathology, orthostasis, and Lyme disease. Infiltrative disease such as cardiac sarcoidosis was not high on the list of differential diagnoses. Despite low suspicion of meaningful respiratory disease and high efforts to maintain high-value care, it was only on further investigation of his few pulmonary nodules that his final diagnosis was reached. It is in these cases that clinicians are reminded to self-check for diagnostic fallacies, like ambiguity effect and prototypical error in this case, before depriving patients of further workup they may ultimately deserve.

Management of CS is now largely standardized in clinical practice. Corticosteroids, either alone or in combination with other immunosuppressants, remain the mainstay of CS treatment. Patients with diagnosed or suspected CS typically receive a three-day pulse of intravenous methylprednisolone followed by prednisone 40 mg/day for a minimum of four weeks [12]. Given the high risks of sudden cardiac death, patients with severe arrhythmia or AV block should also be considered for ICD placement in addition to medical therapy; this is classified as a class IIa recommendation by the 2014 HRS guidelines and is supported by additional studies regarding CS patients with advanced conduction system abnormalities [6,8,14]. Interestingly, two cases were found in which AV block appeared to be reversed by corticosteroid therapy alone. In one case, a patient with complete heart block reversed to second degree AV block Mobitz type II and later to normal AV conduction after four weeks of corticosteroids treatment, and in the second case, a patient with complete heart block reversed to first degree AV block after two months of corticosteroid treatment [18-19]. While unable to be explored in our above case, it may be worth exploring the viability of medical monotherapy as a standalone treatment for CS, which offers the ultimate benefit of alleviating emotional, financial, and logistical burdens of cardiac device placement and its lifetime potential for further complications.

## Conclusions

Cardiac sarcoidosis is an underappreciated phenotype of sarcoidosis. While an expected presentation of sarcoidosis might be in an AA female presenting with respiratory involvement, it is worth noting that males carry a higher risk of CS even though Caucasian males in particular have the least incidence. Cardiac symptoms may be the first and only manifestations of disease, most commonly being AV block. With the help of PET imaging, as well as biopsies if able, CS diagnosis and intervention can be reached early by maintaining hypervigilance for unexpected presentations as above. Further studies should also be pursued regarding the viability of medical monotherapy with corticosteroids for heart block reversal in lieu of intracardiac devices given their high risk for lifetime complications.
